# Modeling of a Robust Confidence Band for the Power Curve of a Wind Turbine

**DOI:** 10.3390/s16122080

**Published:** 2016-12-07

**Authors:** Wilmar Hernandez, Alfredo Méndez, Jorge L. Maldonado-Correa, Francisco Balleteros

**Affiliations:** 1 Departamento de Ciencias de la Computación y Electrónica, Universidad Técnica Particular de Loja, Campus de la Universidad Técnica Particular de Loja, Calle San Cayetano Alto s/n, Loja 1101608, Ecuador; jlmaldonado7@utpl.edu.ec; 2 Departamento de Matemática a las Tecnologías de la Información y Comunicaciones, Universidad Politécnica de Madrid, Av. Complutense Nº 30, 28040 Madrid, Spain; alfredo.mendez@upm.es (A.M.); francisco.ballesteros@upm.es (F.B.)

**Keywords:** SCADA system, power curve, power-curve confidence band

## Abstract

Having an accurate model of the power curve of a wind turbine allows us to better monitor its operation and planning of storage capacity. Since wind speed and direction is of a highly stochastic nature, the forecasting of the power generated by the wind turbine is of the same nature as well. In this paper, a method for obtaining a robust confidence band containing the power curve of a wind turbine under test conditions is presented. Here, the confidence band is bound by two curves which are estimated using parametric statistical inference techniques. However, the observations that are used for carrying out the statistical analysis are obtained by using the binning method, and in each bin, the outliers are eliminated by using a censorship process based on robust statistical techniques. Then, the observations that are not outliers are divided into observation sets. Finally, both the power curve of the wind turbine and the two curves that define the robust confidence band are estimated using each of the previously mentioned observation sets.

## 1. Introduction

Energy consumption is an indicator of the modern development of a society. One consequence of the oil crisis was the need to replace the use of conventional energy sources in the industry with renewable sources. These new forms of alternative energy generation had to possess the ability to pollute the environment as little as possible. In this sense, one of the leading natural resources of alternative energy is the wind. The rapid growth of wind farms worldwide entails the analysis of all elements of the wind turbine in order to ensure its optimum performance, since it is difficult to increase it in size and because of the high economic cost, especially for those installed in steep places or in the sea, where the higher speed of the wind is harnessed. The most influential parameter in wind generation is the wind speed. However, other factors may also be involved, such as the area of the turbine rotor, the air density, the pitch angle, the mechanical characteristics of the turbine, the aerodynamic characteristics of the blades, and the orographic conditions of the location of the wind turbines, among others. However, continuous monitoring of the parameter of wind speed and the electric power generated by the turbine can provide an overview of the performance of the turbine [1]. A brief description of the state of the art, based on an exhaustive review of the scientific literature describing the different types of defects in the turbines and diagnostic schemes, among other things, is given in [2,3,4]. In [5], the authors explained the basic concept of the power curve, the different methodologies that are used for its estimation, and presented an approximation for the estimation of this curve by using a kernel method with multiple factors. Also, the use of the kernel method can be seen in [6].

Here, it is worth mentioning that the power curve of a wind turbine is a parameter that is used to make predictions on the electricity generation of wind farms.

In this paper, a model to estimate the variation of the power generated by a wind turbine under study is presented. Here, the input variable is the wind speed measured by the anemometer which is situated in the nacelle of the wind turbine, and the data used for the analysis (the wind speed and power output information) was taken from the SCADA (Supervisory Control and Data Acquisition) system of the turbine. The most important contribution of this paper is that a model for a robust confidence band of the power curve is presented.

It should be highlighted that this type of modeling allows wind farm operators to carry out the proper maintenance of the turbines, improve their lifetime, adjust parameters of interest, and check the electromechanical performance characteristics that are influencing either the increase or decrease of the performance of the wind turbines.

In previous research works, the frequency response of a wind turbine under assessment was obtained [7], and novel methods of verifying the power performance of both a wind turbine and a wind farm were presented in [8,9].

Other applications of the method developed in this paper in different fields of research are the following: In [10], robust measures are used to study the distribution of Belgian consumer price changes and its interaction with aggregate inflation over the period of June 1976–September 2000. In that paper, robust measures of location, scale, skewness, and tail weight are presented. Such a paper is aimed at studying the cross-sectional properties of Belgian inflation data. In [10], classical characteristics of location, scale, skewness, and kurtosis are compared with robust alternatives for location, skewness and tail weights.

In [11], robust statistical methods are applied to two problems in computer vision: range image fitting and segmentation, and image motion estimation. In [11], the authors looked at characterizing the quality of a model fit by capturing information such as how peaked around zero the residual probability density function (pdf) is. To that end, in [11] procedures that use kernel density estimation of the pdf and a mean-shift approach to locate the peak of that pdf are devised.

In [12], robust statistical techniques are used in mechanical vibration measurements. In that paper, robust regression algorithms are used for laser position calibration, when the vibration response at a large number of locations is desired. Also, quasi-robust processing of vibration measurements is shown and robust modeling techniques for vibration measurements are illustrated.

The above-mentioned references represent a list, by no means complete, of applications of robust statistics to solve industry and technology problems. The present paper shows an application of this type of statistics for modeling a robust confidence band for the power curve of a wind turbine.

The organization of the rest of the paper is as follows: Section 2 presents some techniques for modeling the power curve. Section 3 is devoted to the data and methods that were used for the estimation of the power curve. Section 4 is devoted to the analysis and experimental results. Finally, Section 5 is devoted to the conclusions of this paper.

## 2. Techniques for Modeling the Power Curve

The power supplied by a wind turbine is often represented by its power curve, where a relationship between the wind speed and power generated is given by [13]

(1) $$p\;=\frac{1}{2}\rho AC_{p}v^{3},$$

where *p* is the power, ρ is the air density, *A* is the rotor area, $C_{p}$ is the power coefficient, and *v* is the wind speed.

At this point it is important to point out that, in accordance with [5,13], the power coefficient is believed to be a function of, at least, the blade pitch angle and the tip-speed ratio of the turbine. Nevertheless, research into how other variables affect the power coefficient is still open for investigation. At present, the power coefficient is empirically estimated and turbine manufacturers provide, for a specific turbine, its nominal power curve with its corresponding power coefficient values under different combinations of wind speed and air density.

Considering this theoretical model given by Equation (1), anomalies of wind turbines regarding random variation of the generated power in relation to the wind speed cannot be incorporated. Many studies have been focused on this issue and various models have been used to describe the variation of wind energy in stochastic terms. A first constraint is to express the power curve represented as follows:

(2) $$p\left(v\right)=\{\begin{array}{c} \begin{array}{cc} \begin{array}{c} 0\\ q\left(v\right)\\ P_{r} \end{array} & \begin{array}{c} v<v_{0}\\ v_{0}\leq v\leq v_{r}\\ v_{r}<v\leq v_{1} \end{array} \end{array}\\ \begin{array}{cc} 0 & v>v_{1} \end{array} \end{array},$$

where $v_{0}$ and $v_{1}$ are the cut-in wind speed and the cut-out wind speed, respectively. Also, $v_{r}$ is the rated wind speed, $P_{r}$ is the rated wind power, and *q*(*v*) is the non-linear relationship between power and wind speed.

In [5], the authors presented a kernel method for a multiple-input/single-output problem. In the multiple-input problem, the authors had to devise a special structure for their kernel method to handle the challenge. The kernel method was able to handle it satisfactorily (see Jeon and Taylor [6]), and this method can be applied to the single-input problem, as well.

Polynomial regression methods are techniques widely used to adjust the power curve. In [13], models of *q*(*v*) are presented as polynomial and exponential curves. Estimation using polynomial regression, polynomial regression by weighing, and spline have also been used in [14]. Regression procedures have been used by using logistic equations in [15], also proposing models by using neural network techniques.

At this point, it is important to highlight some of the conclusions reached by the authors of [15]. In short, in [15], parametric and nonparametric models of wind turbine power curves were developed. The parametric models were the following: the linearized segmented model, four-parameter logistic expression, and five-parameter logistic expression. On the other hand, in [15], nonparametric models were developed using neural networks, the fuzzy c-means clustering algorithm, and data mining algorithms.

Also, the modeling techniques used in [15] for the development of parametric and nonparametric models of wind turbine power curves were the following:

(1) Techniques for parametric models: modeling of the power curve using the least squares method, modeling of the power curve using genetic algorithms, modeling of the power curve using evolutionary programming, modeling of the power curve using particle swamp optimization and modeling of the power curve using differential evolution.
(2) Techniques for nonparametric models: modeling of the power curve using neural networks, modeling of the power curve using fuzzy c-means clustering, and modeling of the power curve by using four data-mining algorithms (i.e., bagging, the M5P algorithm, the REPTree algorithm, and M5Rules).

One interesting conclusion of [15] was that when working with real-time datasets, parametric models perform better than nonparametric models. From our point of view, this conclusion is in agreement with what statistics teaches us: if the random variables under analysis follow a parametric distribution, parametric techniques perform better than nonparametric techniques.

In [15], the application of the differential evolution algorithm to a five-parameter logistic function gave the best parametric model of a wind turbine power curve, and the neural network algorithm gave the best nonparametric model.

In order to approximate the behavior of the power curve, models based on data mining have also been used in [16] by groups using cluster center fuzzy logic models, neural network models, and *k-*nearest neighbor models. In addition, a comparison with adaptive neuro-fuzzy interference system models is carried out. The main contribution of [16] is that the authors of that paper carried out direct comparisons of different model approaches found in the literature, based on datasets reflecting modern turbine behavior (pitch-regulated turbines).

The authors of [16] concluded that next to wind speed, the ambient temperature and the wind direction are important parameters when setting up data-mining models for wind turbine power curve monitoring. Furthermore, in [16], it is concluded that the performance of adaptive neuro-fuzzy interference system models that are enhanced by considering the ambient temperature and the wind direction was the best.

In [17], it is proposed that Gaussian models and censored Gaussian models are used to approximate the power curve. In [18], the authors presented a wind power forecasting model that consisted of the Gaussian process with a novel composite covariance function for high-accuracy wind power forecasting. The composite covariance function of [18] was based on the exploration of joint effects between numerical weather prediction features.

Irregularities often occur, and when an irregularity occurs, part of the power generated is lost. These anomalies, which appear on the power curve, have been discussed in [19] to try to relate the probability of the interruption of the operation of wind turbines with the wind speed. Also, a model of energy production in the frequency domain is proposed in [19]. The problem of the appearance of outliers in the model has been analyzed by using heavy-tailed distributions when there are significant changes in wind speed [20]. In order to eliminate possible outliers, robust filtering techniques have been used to determine the deviation of the turbine performance. These techniques can be found in [21]. In [22], the Copula theory is adopted to establish the probability distribution of correlated input random variables, and this theory is also applied for constructing the multivariate distribution function of wind speeds at different wind sites [23].

## 3. Data and Methods Used for the Estimation of the Power Curve

**Figure 1:**
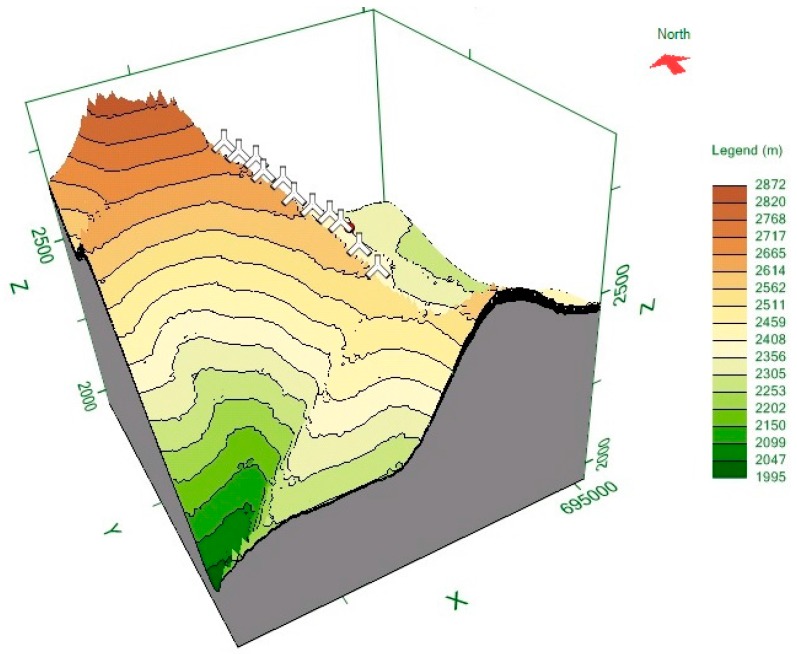
Orographic map of the Villonaco Wind Farm with wind turbine positions in UTM coordinates.

**Figure 2:**
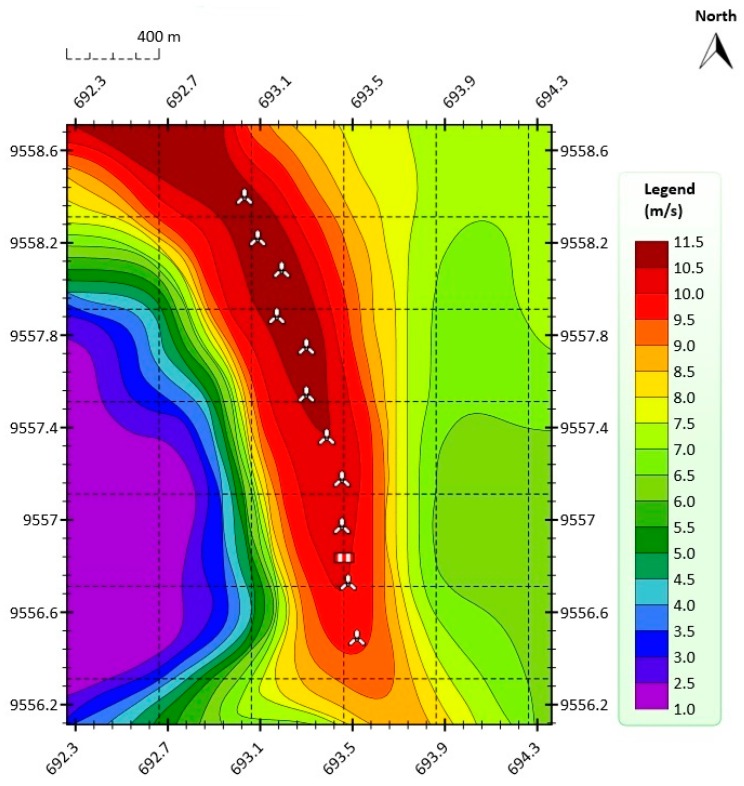
Annual average wind speed at 100 m AGL at the VWF in the year 2014.

The wind turbine under study in the present paper is placed in a complex terrain [24,25,26]. This turbine is placed in the Villonaco Wind Farm (VWF), Loja, Ecuador [7,8,9,27]. The Universal Transverse Mercator (UTM) coordinate system of this wind turbine is: 693.035 East and 9558.399 North. In addition, the elevation of this turbine is 2753.4 m above sea level. The VWF consists of 11 *×* 1.5 MW Goldwind GW70, Permanent Magnet Direct Drive, IEC Class “S” wind turbine generators along a ridge approximately 6 km (aerial distance) to the west of the city of Loja. Figure 1 shows the orographic map of the VWF. Figure 2 shows the annual average wind speed at 100 m above ground level (AGL) at the VWF. The annual mean wind at 100 m AGL was greater than 10.5 m/s in the year 2014, which was the year the data were collected (from 1 January 2014 to 31 December 2014).

In this paper, in order to obtain the orographic map shown in Figure 1 and the annual average wind speed map shown in Figure 2, the Meteodyn WT wind resource assessment software was used [28,29,30]. Here, the input variables used were the following: topographic and roughness data of the VWF; wind speed data of the meteorological tower located in the VWF; guaranteed power curve of the WTs; air density of the VWF (0.923 kg/m^3^); hub height of the WTs; wind turbine positions in UTM coordinates; and meteorological tower position in UTM coordinates.

**Figure 3:**
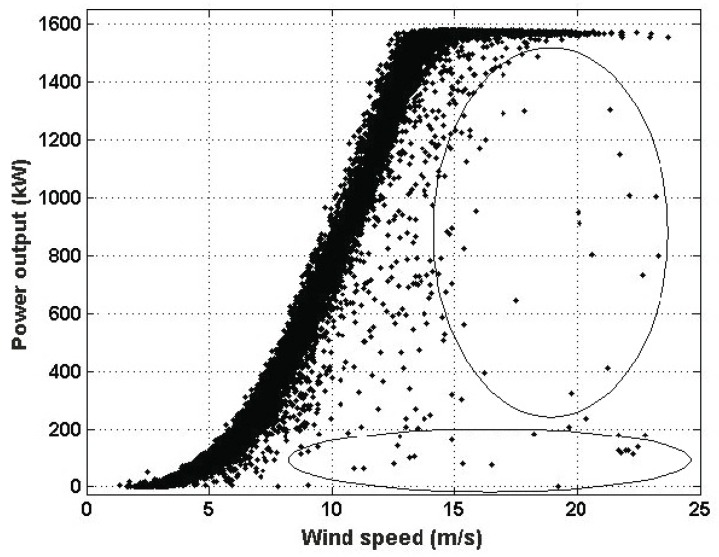
Power curve in which some of the anomalous data are marked.

As was previously mentioned, the time measurement interval was from 1 October 2014 to 31 December 2014, the information about the wind speed and power output was taken from the Goldwind SCADA system, the hub height was equal to 65 m, and the sampling interval was equal to 10 min [7,8,9,31]. Figure 3 shows the scatter plot of the power output against the wind speed (i.e., the power curve) identifying some of the points ($p_{i}$, $v_{i}$) that are affected by any anomaly.

Here, regression models are used to explain the behavior of the power output based on the behavior of the wind speed. Also, it is assumed that the power output can be expressed as *p* = *f*(*v*) + *u*, where *f* is the function that depends on the predictor *v* which we want to find, and the effect of the other variables affecting the model is grouped into a sum, *u*, which is called the error term. In establishing the model it is assumed that errors behave like a normal distribution with zero mean and constant variability, and that they are uncorrelated. Because of the assumptions that have been imposed on the model, it is going to be verified that the power output *p*, in terms of the mean, is determined by *f*(*v*), and that the term, including the other effects that have an impact on the power output, has constant variability.

In this research, the theoretical model in Equation (1) is estimated, and also several power curves are estimated by using different functions in order to adjust *q*(*v*) by the regression method. These functions are the following: a second-degree polynomial function (see Equation (3)), an exponential function (see Equation (4)), and a Gaussian function (see Equation (5)),

(3) $$q\left(v\right)=C_{2}v^{2}+C_{1}v+C_{0},$$

(4) $$q\left(v\right)=\frac{1}{2}\rho AK_{p}\left(v^{\beta }-{v_{1}}^{\beta }\right),$$

(5) $$q\left(v\right)=L\cdot e^{-\frac{1}{2}{\left(\frac{v-u}{\sigma }\right)}^{2}},$$

where $C_{0}$, $C_{1}$ and $C_{2}$ depend on $v_{1}$ and $v_{r}$ (see Equation (2)), and $K_{p}$ and β are constants. These functions are used because the shape of the graphical representations of the curves is very similar to the shape of the curve around which the data are grouped.

In the analysis that is made in this paper, piecewise estimations of power curves are compared, making sure that all models have a high rate of adjustment, although with very different expressions, and that the differences between them are, in some areas, negligible. Then, once the models are adjusted, the residuals are analyzed. If the model is correct, each residual is an estimate of the error and therefore the analysis of residuals is used a posteriori for testing the assumptions made on the model. It will be shown that the behavior of the residual variance is a non-constant behavior and that it depends on the wind speed.

The observed anomalies can be considered outliers [32]. For distributions such as the present one, an outlier is considered to be a kind of observation that breaks the model. In other words, the outliers are those observations a random variable which under certain methodologies are supposed to be incorrect data. In this research, due to the fact that there is a large number of observations that appear outside of the curve on which observations are grouped together, robust statistical methods [33] are going to be applied because these methods are less susceptible to variability of the data than the conventional methods, in which all the observations are considered.

## 4. Analysis and Experimental Results

In order to carry out the analysis, numerical values for which the power output was less than or equal to zero were discarded, because these values represent faults in the performance of the turbine during its operation. Then, the binning method was used, the data were grouped into wind speed intervals of a length of 0.2 m/s in order to obtain the data grouped into *l* classes, considering that for each class there must exist enough observations, $m_{i}$, *i* = 1,…,*l*. Next, to obtain robust confidence intervals in each group, a robust estimation of a location parameter and of a scale parameter was made. The location parameter was the median ($Me_{i}$), and the scale parameter was the interquartile range ($IQR_{i}$). The robust confidence intervals (CI) were calculated by using

(6) $$CI_{\left[1-\propto \right],\;k}\left(i\right)=\left(Me_{i}\pm k\cdot \frac{IQR_{i}}{\sqrt{m_{i}}}\right),$$

where the real number *k* is a constant that was chosen such that the set of all observations that fall within all the confidence intervals was greater than the 100·(1−∝)% of the observations.

**Figure 4:**
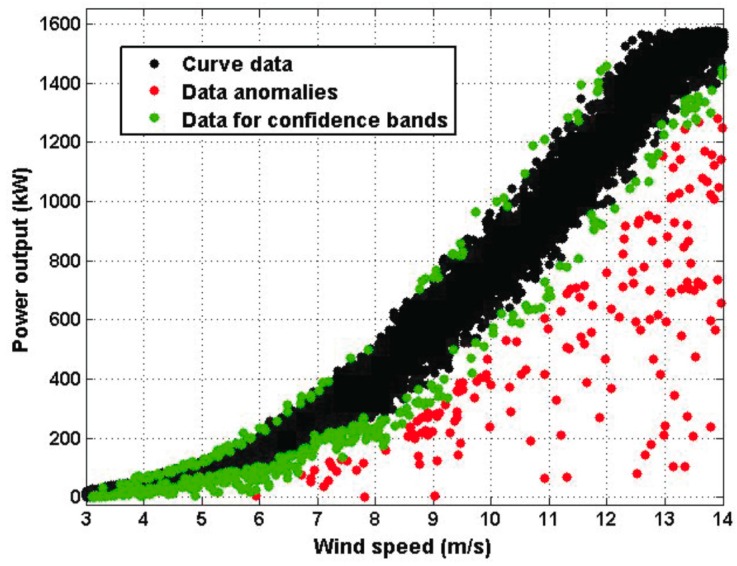
Robust classification of the data in the wind speed interval [3 m/s, 14 m/s].

In this paper, the observations that did not fall within the confidence intervals were excluded and observations that did fall within these intervals were used to establish the model. In addition, the data that was not excluded was separated based on its distance with respect to the tolerance limits. Thus, unsuppressed data that fell within the intervals

(7) $$CI_{\left[1-\propto \right],\;k-h}\left(i\right)=\left(Me_{i}\pm \left(k-h\right)\cdot \frac{IQR_{i}}{\sqrt{m_{i}}}\right),$$

where the real number *h* is a constant that was chosen to be 0 *< h < k*, which were the ones used to estimate the model, and the unsuppressed data that fell outside of Equation (7) were the ones used to estimate the limits of the confidence band. Furthermore, *k* and *h* were chosen to ensure that for the estimation process, 98% of the observations were used by using Equation (6), and 92% of the observations were used by using Equation (7). Figure 4 shows the robust classification of the data that was used to estimate both the model and the limits of the confidence band in the range of wind speed values from 3 m/s up to 14 m/s. In Figure 4, the black points represent 92% of the data, the green points represent 6% of the data, and the red points represent points that fell outside the interval in Equation (6).

The function *q*(*v*) of Equation (2) was adjusted by using Equations (1), (3)–(5). Also, a line segment was used to approximate the power curve in the region below $v_{0}$. Next, using the central curve and the line segment, the model was completed by continuity,

(8) $$p\left(v\right)=\{\begin{array}{cc} 0 & v<2.2\;m/s\\ q\left(v\right)= & \{\begin{array}{cc} \begin{array}{c} a\cdot v+b\\ f\left(v\right) \end{array} & \begin{array}{c} 2.2\;m/s\leq v\leq 3\;m/s\\ 3\;m/s\leq v\leq 14\;m/s \end{array} \end{array}\\ \begin{array}{c} P_{r}\\ 0 \end{array} & \begin{array}{c} 14\;m/s<v\leq 23.75\;m/s\\ v>23.75\;m/s \end{array} \end{array},$$

where $P_{r}$ is a constant number.

In order to measure the goodness of fit of the statistical model, the coefficient of determination (*R*^2^) and the root-mean-square error (RMSE) were used. Further, 100·*R*^2^ represents the percentage of the variability of the response that is explained by the model. Table 1 shows the estimated models for the power output in the wind speed interval [3 m/s, 14 m/s].

**Figure 5:**
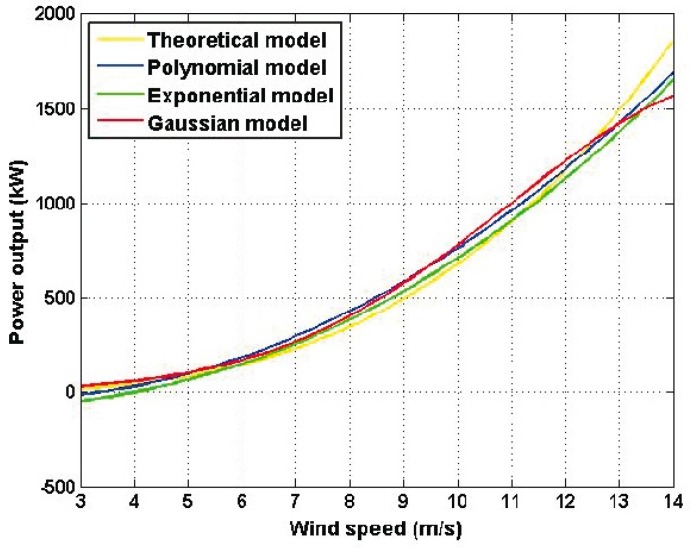
Estimated models of Table 1: Theoretical model (Equation (1)); Polynomial model (Equation (3)); Exponential model (Equation (4)); and Gaussian model (Equation (5)).

From Table 1, it can be seen that the estimated models have very different mathematical expressions, but the *R*^2^ values are very high and the RMSE values are low. Figure 5 shows the four estimated models. Despite the difference in the expressions of the models in Table 1, Figure 5 shows that their graphs are quite similar. Perhaps the graph representing the theoretical model of Equation (1) moves away from the other graphs a little bit; also the *R*^2^ value and RMSE value for that model represented the poorest performance (see Table 1).

**Figure 6:**
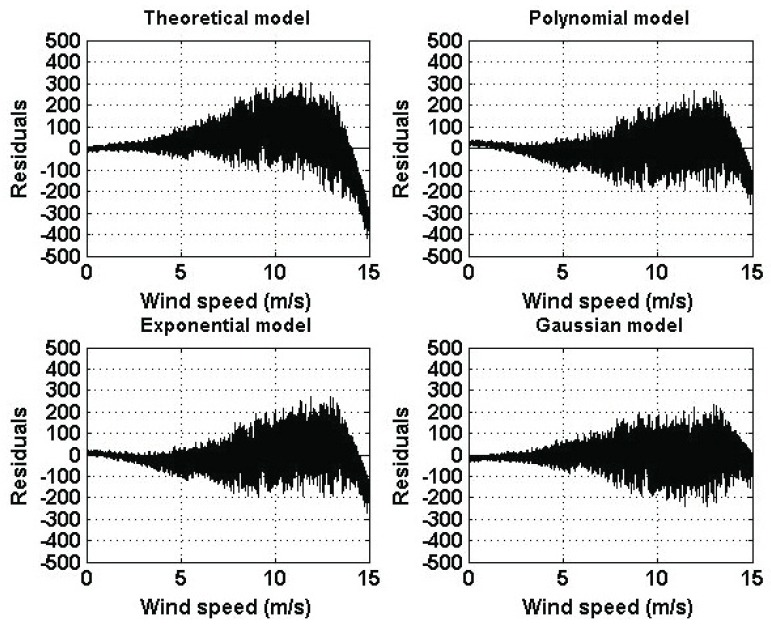
Residuals of the estimated models of Table 1.

**Figure 7:**
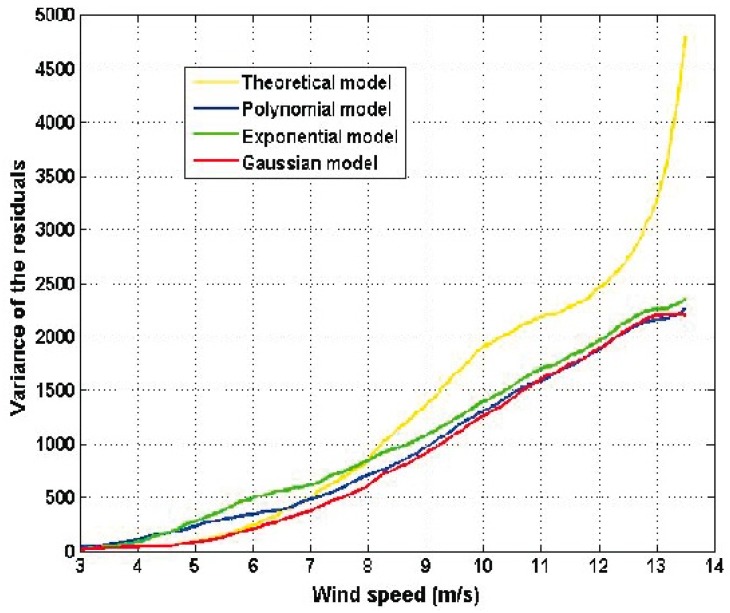
Variance of the residuals of the estimated models of Table 1.

Figure 6 shows the residuals of the estimated models and Figure 7 shows the variance of the residuals.

As a result of the similarity among the graphs shown in Figure 5, the residuals are similar in their form. Also, it can be seen that the error term has a variability that increases as the wind speed increases. Furthermore, residual variances are similar in terms of form and increase as the wind speed increases. Again, the residuals of the theoretical model are greater than the residuals of the other models. Moreover, the residual variance of the theoretical model is significantly greater than the variances of the other models. Therefore, assuming that the theoretical model is correct, there must exist a wind speed that compensates for $v^{3}$ in Equation (1) and the only place in Equation (1) where such a wind speed can be introduced is in the power coefficient, $C_{p}$. For this reason, based on the experimental results, the authors of this paper believe that $C_{p}$ depends on the wind speed. The latter comment is also made by the authors of [5,13]. For the actual wind turbine under study, the Gaussian model is the best one.

For a confidence band containing at least 90% of the observations, the selected points that were close to the tolerances that were used to delete data (see Equation (6)) were considered. The Gaussian model was chosen for the power curve in the wind speed interval [3 m/s, 14 m/s]. The confidence band for the Gaussian model was given by

(9) $$1826\cdot e^{-{\left(\frac{v-17.17}{6.44}\right)}^{2}}\leq q\left(v\right)\leq 1602\cdot e^{-{\left(\frac{v-13.94}{6.63}\right)}^{2}},$$

**Figure 8:**
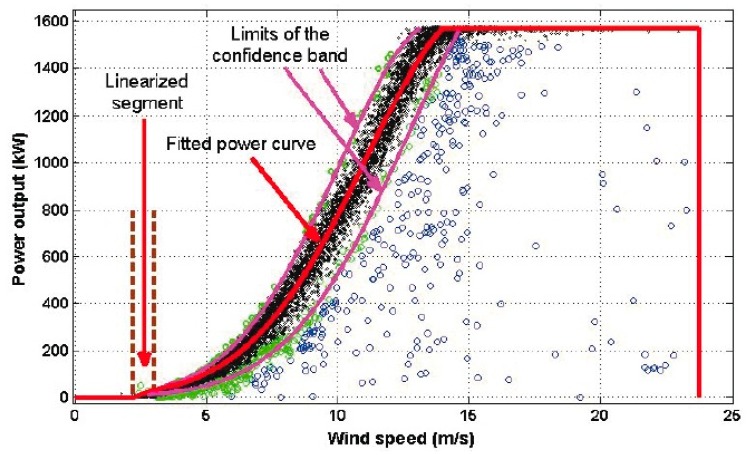
Confidence band for the power curve. Curve data: black; Data used to estimate the confidence band: green; Data anomalies: blue.

Figure 8 shows the limits of the confidence band (i.e., the curve of high production efficiency and the curve of low production efficiency) and the estimated power curve (see Equation (9)). For this case, *a* = 40.6225, *b* = *−*88.5757, and $P_{r}$ = 1570 kW (see Equation (8)). In addition, all models estimated in this paper fell within this confidence band, and 93.8% of the power output points for the wind speed interval [3 m/s, 14 m/s] fell within this confidence band as well.

At this point, it is important to point out that in [5], the authors showed a graph in Figure 1 of their paper which is an example of a power curve that, in some way, is similar to Figure 8 of the present paper. However, Figure 1 of [5] is shown only as a general example of the form of the power curve, which also has been adjusted on purpose by the authors of [5] with the aim of giving the reader only a cursory visual idea of the form of the high and low production efficiency curves. Nevertheless, in the research work carried out in this paper, a methodology to obtain a robust confidence band is presented. Furthermore, here it is guaranteed that the robust confidence band belongs to a parametric family, and that this band contains more than 90% of the observations.

## 5. Conclusions

In this paper, taking into account the data that was considered, a model to estimate the variation of the power generated by a wind turbine in terms of wind speed has been proposed. Here, robust methodologies have been used to select a portion of the observations in order to conduct the statistical analysis. Then, the estimated power of the wind turbine against the wind speed was represented.

Here, four models used to model the power curve of a wind turbine have been analyzed. These models consisted of a function that is based on the pure physics of wind turbines, a second-degree polynomial function, an exponential function, and a Gaussian function. Also, in order to measure the goodness of fit of the statistical models, the coefficient of determination and the root-mean-square error were used. After analyzing the different adjustment models that were used in this research, it was found that despite the difference in the expressions of the four models, their graphs were quite similar, especially those of the polynomial model, the exponential model, and the Gaussian model. Perhaps the graph representing the theoretical model moved away from the other graphs a little bit. In addition, the coefficient of determination and the root-mean-square error of that model represented the poorest performance.

Furthermore, as a result of the similarity among the graphs of the four models, the residuals were similar in their form. The residuals indicated that these models are models with heteroscedasticity. Moreover, it was found that the error term has a variability that increases as the wind speed increases, and that the residual variances also increase as the wind speed increases. The residuals of the theoretical model were greater than the residuals of the other models, and the residual variance of the theoretical model was significantly greater than the variances of the other models.

For the actual wind turbine under study, it was found that the Gaussian model was the one with the best performance. In addition, it is worth mentioning that, assuming that the theoretical model is correct, based on the experimental results, the authors of this paper believe that the power coefficient depends on the wind speed. As was mentioned in the body of this paper, other authors cited in the references arrived at the same conclusion.

The most important contribution of this research is that a model to obtain a robust confidence band of the power curve of the wind turbine under study has been presented. This model is important because it explains the behavior of more than 90% of the observations in the range of wind speed between the cut-in wind speed and the cut-out wind speed. Modeling the power curve of wind turbines in an accurate and realistic manner is of paramount importance for wind farm owners and operators, because this curve is used to evaluate the performance of the turbines and to make predictions about the electricity generation of wind farms.

## Figures and Tables

**Table 1:** Estimated models for the power output.

Models	*f*(*v*)	*R^2^*	RMSE
Equation (1)	$0.68\cdot v^{3}$	0.9653	92.46
Equation (3)	$11.03\cdot v^{2}-32.63\cdot v-14.22$	0.9850	60.82
Equation (4)	$4.07\cdot \left(v^{2.157}-3^{2.157}\right)$	0.9858	62.71
Equation (5)	$1655\cdot e^{-{\left(\frac{v-15.44}{6.27}\right)}^{2}}$	0.9877	55.02

## References

[1] Kusiak A., Verma A. (2013). Monitoring Wind Farms with Performance Curves. IEEE Trans. Sustain. Energy.

[2] Garcia-Marquez F.P., Tobias A.M., Pinar-Perez J.M., Papaelias M. (2012). Condition Monitoring of Wind Turbines: Techniques and Methods. Renew. Energy.

[3] Hameed Z., Hong Y.S., Cho Y.M., Ahn S.H., Song C.K. (2009). Condition Monitoring and Fault Detection of Wind Turbines and Related Algorithms: A Review. Renew. Sustain. Energy Rev..

[4] Amirat Y., Benbouzid M.E.H., Al-Ahmar E., Bensaker B., Turri S. (2009). A Brief Status on Condition Monitoring and Fault Diagnosis in Wind Energy Conversion Systems. Renew. Sustain. Energy Rev..

[5] Lee G., Ding Y., Genton M.G., Xie L. (2015). Power Curve Estimation With Multivariate Environmental Factors for Inland and Offshore Wind Farms. J. Am. Stat. Assoc..

[6] Jeon J., Taylor J.W. (2012). Using Conditional Kernel Density Estimation for Wind Power Density Forecasting. J. Am. Stat. Assoc..

[7] Hernandez W., Maldonado-Correa J.L., Mendez A. (2016). Frequency-domain Analysis of Performance of a Wind Turbine. Electron. Lett..

[8] Hernandez W., Lopez-Presa J.L., Maldonado-Correa J.L. (2016). Power Performance Verification of a Wind Farm by Using the Friedman’s Test. Sensors.

[9] Hernandez W., Maldonado-Correa J.L. (2016). Power Performance Verification of a Wind Turbine by Using the Wilcoxon Signed-Rank Test. IEEE Trans. Energy Convers..

[10] Aucremanne L., Brys G., Hubert M., Rousseeuw P.J., Struyf A., Hubert M., Pison G., Struyf A., Van Aelst S. (2004). A Study of Belgian Inflation, Relative Prices and Nominal Rigidities using New Robust Measures of Skewness and Tail Weight. Theory and Applications of Recent Robust Methods.

[11] Suter D., Wang H., Hubert M., Pison G., Struyf A., Van Aelst S. (2004). Robust Fitting Using Mean Shift: Applications in Computer Vision. Theory and Applications of Recent Robust Methods.

[12] Vanlanduit S., Guillaume P., Hubert M., Pison G., Struyf A., Van Aelst S. (2004). Robust Processing of Mechanical Vibration Measurements. Theory and Applications of Recent Robust Methods.

[13] Carrillo C., Obando Montano A.F., Cidra J., Diaz-Dorado E. (2013). Review of Power Curve Modelling for Wind Turbines. Renew. Sustain. Energy Rev..

[14] Shokrzadeh S., Jozani M.J., Bibeau E. (2014). Wind Turbine Power Curve Modeling Using Advanced Parametric and Nonparametric Methods. IEEE Trans. Sustain. Energy.

[15] Lydia M., Selvakumar A.I., Kumar S.S., Kumar G.E.P. (2013). Advanced Algorithms for Wind Turbine Power Curve Modeling. IEEE Trans. Sustain. Energy.

[16] Schlechtingen M., Santos I.F., Achiche S. (2013). Using Data-Mining Approaches for Wind Turbine Power Curve Monitoring: A Comparative Study. IEEE Trans. Sustain. Energy.

[17] Chen N., Qian Z., Nabney I.T., Meng X. (2014). Wind Power Forecasts Using Gaussian Processes and Numerical Weather Prediction. IEEE Trans. Power Syst..

[18] Fang S., Chiang H.-D. (2016). A High-Accuracy Wind Power Forecasting Model. IEEE Trans. Power Syst..

[19] Cheng L., Lin J., Sun Y.Z., Singh C., Gao W.Z., Qin X.M. (2012). A Model for Assessing the Power Variation of a Wind Farm Considering the Outages of Wind Turbines. IEEE Trans. Sustain. Energy.

[20] Ganger D., Zhang J., Vittal V. (2014). Statistical Characterization of Wind Power Ramps via Extreme Value Analysis. IEEE Trans. Power Syst..

[21] Sainz E., Llombart A., Guerrero J.J. (2009). Robust Filtering for the Characterization of Wind Turbines: Improving its Operation and Maintenance. Energy Convers. Manag..

[22] Cai D., Shi D., Chen J. (2014). Probabilistic Load Flow Computation Using Copula and Latin Hypercube Sampling. IET Gener. Transm. Distrib..

[23] Xie K., Li Y., Li W. (2012). Modelling Wind Speed Dependence in System Reliability Assessment Using Copulas. IET Renew. Power Gener.

[24] Polanco G., Virk M.S. Role of Advanced CAE Tools in the Optimization of Wind Resource Assessment of Complex Terrains. Proceedings of the 4th IEEE International Conference Cognitive Infocommunications (CogInfoCom 2013).

[25] Cao Y., Li H., Lu Z., Wang W. The Operating Risk Evaluation of Power System with a Large Scale of Wind Farms Considering Uncertainty of Wind Power Prediction and Extreme Weather. Proceedings of the 2nd IET Renewable Power Generation Conference (RPG 2013).

[26] Zhang H., Ou H., Zhang Y., Zhu X. Research on the Wind Resource Assessment in Mountainous Complex Terrains Based on ArcGIS. Proceedings of the 2nd IET Renewable Power Generation Conference (RPG 2013).

[27] Robalino-Lopez A., Mena-Nieto A., Garcia-Ramos J.E. (2014). System Dynamics Modeling for Renewable Energy and CO_2_ Emissions: A Case Study of Ecuador. Energy Sustain. Dev..

[28] Meteodyn WT: Help Facility and On-Line Documentation. http://www.meteodyn.com.

[29] Promsen W., Masiri I., Janjai S. (2012). Development of Microscale Wind Maps for Phaluay Island, Thailand. Procedia Eng..

[30] Pereira R., Guedes R., Silva Santos C. Comparing WAsP and CFD Wind Resource Estimates for the “Regular” User. http://meteodyn.com/wp-content/uploads/2012/05/Comparing-WAsP-and-Meteodyn-wind-resource-estimates-for-the-regular-user_paper.pdf.

[31] IEC 61400-12-1: Wind Turbines—Part 12-1: Power Performance Measurements of Electricity Producing Wind Turbines. https://webstore.iec.ch/preview/info_iec61400-12-1%7Bed1.0%7Den.pdf.

[32] Barnet V., Lewis T. (1994). Outliers in Statistical Data.

[33] Hoaglin D.C., Mosteller F., Tukey J.W. (1983). Understanding Robust and Exploratory Data Analysis.

